# Pharmacologic evaluation of shivering management in neurologically injured patients utilizing therapeutic normothermia

**DOI:** 10.1186/cc14507

**Published:** 2015-03-16

**Authors:** T Lam, B Heather, J Jancik

**Affiliations:** 1 Hennepin County Medical Center, Minneapolis, MN, USA

## Introduction

Uncontrolled shivering may have negative consequences by increasing metabolic demand and subsequently neutralize the benefits of therapeutic normothermia [1]. Previous anti-shivering protocols that utilize the least sedation have been described in therapeutic temperature modulation (TTM) [2]. Our aim is to describe and evaluate an anti-shivering protocol that emphasizes the least sedating regimen with the least number of pharmacologic agents for patients undergoing therapeutic normothermia.

## Methods

This retrospective chart review includes patients with neurologic injury who underwent TTM from March 2013 to November 2014 and were evaluated for the following outcomes: percentage of total patient hours in each score of the Bedside Shivering Assessment Scale (BSAS) at 72 hours, 168 hours, and total duration of TTM; percentage of total patient days in each tier of the anti-shivering protocol at 72 hours, 168 hours, and total duration of TTM; de-escalation of anti-shivering agents with or without the necessary need for re-escalation; ICU and hospital length of stay (LOS); rescue agents utilized; and hospital mortality.

## Results

Evaluation of 47 patients who underwent TTM resulted in a total of 505 patient-days of TTM with 6,967 BSAS hours. Overall, patients spent 85.5% of total hours at BSAS 0, 11.4% of total hours at BSAS 1, 2.5% of total hours at BSAS 2, and 0.6% of total hours at BSAS 3. Patients were in tier 0 of the anti-shivering protocol 33.1% of the time, in tier 1 of the anti-shivering protocol 20.6% of the time, in tier 2 of the anti-shivering protocol 43.6% of the time, and in tier 3 of the anti-shivering protocol 2.8% of the total duration of TTM. There were 487 rescue doses of fentanyl and 243 rescue doses of meperidine that were required for shivering. Patients had a mean ICU LOS of 19 days, mean hospital LOS of 21 days, and a mortality rate of 23.4%.

## Conclusion

This study demonstrates a high level of efficacy of our protocol and the feasibility of de-escalation to limit the number of pharmacologic interventions. With our patient population spending a large percentage of time without shivering, it would suggest that this protocol could be revised further by utilizing rescue agents more frequently in order to prevent escalation of therapy to the next tier.

## References

[B1] Badjiatia N Crit Care Med 2009 37 S250 7 10.1097/CCM.0b013e3181aa5e8d 19535955

[B2] Choi HA Neurocrit Care 2011 14 389 94 10.1007/s12028-010-9474-7 21210305

